# Supplementary material for: Therapeutic Aqueous Humor Concentrations of Latanoprost Attained in Rats by Administration in a Very-High-Molecular-Weight Hyaluronic Acid Eye Drop
Source: Pharmaceutics. 2024 Apr 9;16(4):523. doi: 10.3390/pharmaceutics16040523 (PMC11053993; doi:10.3390/pharmaceutics16040523)
Supplement: Supplementary file 1 [file pharmaceutics-16-00523-s001.zip › pharmaceutics-2860034-supplementary.pdf]

A Male

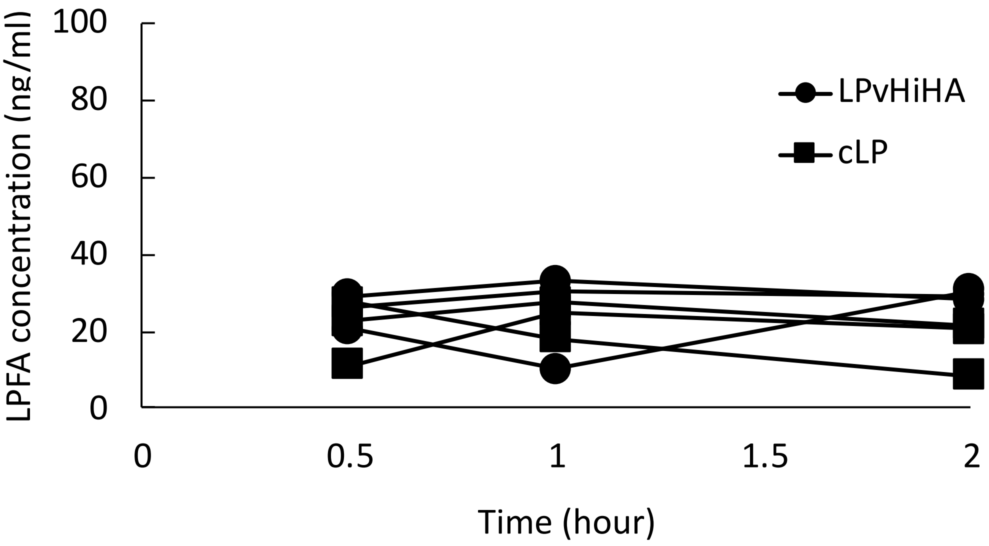

B Female

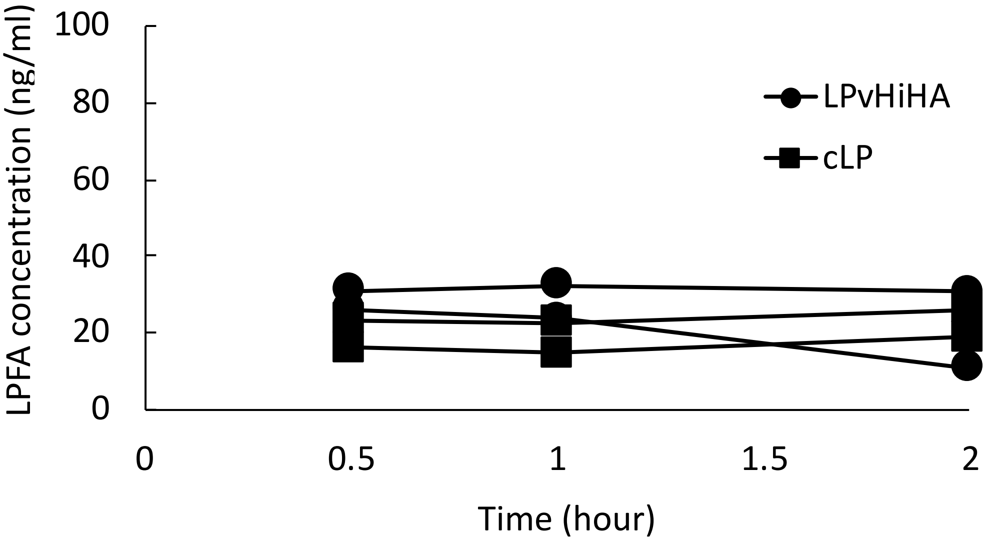

Supplemental Figure S1: The time-dependent changes in LPFA concentration in the rat AH of the untreated contralateral eyes.

Supplemental Table S1.

Mesurement data of Latanoplost free acid (LPFA) concentration (ng/ml) in aqueous humor (AH) of contralateral non-treated eyes as a quality control.

Time (h)	Quality control									
	Male						Female			
	#1	#2	#3	#4	#5	#6	#1	#2	#3	#4
0.5	26.13	29.30	20.41	11.11	22.69	27.55	31.17	26.13	16.09	23.50
1	30.09	33.15	9.99	25.00	27.31	18.20	32.57	23.92	14.99	22.89
2	29.30	28.29	30.62	20.59	21.71	8.38	30.62	10.91	19.02	26.36
